# The Potential Role of Cardiac CT in the Evaluation of Patients With Known or Suspected Cardiomyopathy: From Traditional Indications to Novel Clinical Applications

**DOI:** 10.3389/fcvm.2021.709124

**Published:** 2021-09-14

**Authors:** Edoardo Conte, Saima Mushtaq, Giuseppe Muscogiuri, Alberto Formenti, Andrea Annoni, Elisabetta Mancini, Francesca Ricci, Eleonora Melotti, Carlo Gigante, Zanotto Lorenza, Marco Guglielmo, Andrea Baggiano, Riccardo Maragna, Carlo Maria Giacari, Corrado Carbucicchio, Valentina Catto, Mauro Pepi, Daniele Andreini, Gianluca Pontone

**Affiliations:** ^1^Centro Cardologico Monzino, Istituto di Ricerca e Cura a Carattere Scientifico (IRCCS), Milan, Italy; ^2^Department of Biomedical Science for Health, University of Milan, Milan, Italy; ^3^Department of Clinical Sciences and Community Health, Cardiovascular Section, University of Milan, Milan, Italy

**Keywords:** cardiac computed tomographic imaging, cardiomyopathies, myocardial fibrosis, multimodality imaging, cardiac imaging and diagnostics

## Abstract

After 15 years from its advent in the clinical field, coronary computed tomography (CCTA) is now widely considered as the best first-step test in patients with low-to-moderate pre-test probability of coronary artery disease. Technological innovation was of pivotal importance for the extensive clinical and scientific interest in CCTA. Recently, the advent of last generation wide-coverage CT scans paved the way for new clinical applications of this technique beyond coronary arteries anatomy evaluation. More precisely, both biventricular volume and systolic function quantification and myocardial fibrosis identification appeared to be feasible with last generation CT. In the present review we would focus on potential applications of cardiac computed tomography (CCT), beyond CCTA, for a comprehensive assessment patients with newly diagnosed cardiomyopathy, from technical requirements to novel clinical applications.

## Introduction

Multimodality imaging has recently gained a pivotal role in the management of cardiovascular disease, and the diagnostic work-up of cardiomyopathies, in order to define the different phenotypes, was deeply influenced by the recent introduction of advanced cardiovascular imaging modalities, such as cardiac magnetic resonance (CMR) (1). Even if transthoracic echocardiography (TTE) remains the first step test thanks to its wide availability, it could be limited by the low-image quality and by the lack of tissue characterization. On the contrary, CMR enables reproducible evaluation of biventricular volumes and systolic function together with non-invasive tissue characterization providing pivotal diagnostic insights, especially when TTE is negative for structural heart disease (SHD). In this regard, Andreini et al. in a population of 946 patients with ventricular arrhythmias without pathological findings at TEE identified 241 patients (25.5%) with SHD at CMR (2). These findings have deep clinical consequences because, beyond the accurate identification of structural heart abnormalities, tissue characterization by CMR, thanks to the identification of myocardial fibrosis at late-gadolinium enhancement (LGE) images, is associated with worse cardiovascular prognosis (3, 4). These data support the growing role that CMR gained in recent clinical guidelines for the diagnosis and management of cardiomyopathies (5) and the recent introduction of T1 and T2 mapping techniques may further expand its clinical application (6). However, clinical use of CMR could be limited by resource availability and by some conditions that represent relative or absolute contraindications (7). Moreover, image quality could be affected by metallic elements and cardiac arrhythmias, limiting CMR application in a specific clinical setting; last but not least, claustrophobia may limit access to the CMR environment for some subjects.

In the DANAMI-3-DEFER CMR substudy (8), 181 of 990 eligible patients (18.2%) were excluded from the study due to claustrophobia or contraindication to CMR. Taking into consideration the importance of non-invasive tissue characterization for an up-to-date diagnosis and management of cardiomyopathies, cardiac CT (CCT) recently emerged as a potential alternative to CMR for both the biventricular function evaluation and myocardial fibrosis identification, beyond the well-validated and accepted role for the non-invasive evaluation of coronary anatomy.

The present review will focus on potential applications of CCT for a comprehensive assessment of patients with newly diagnosed cardiomyopathy, from the well-validated evaluation of coronary anatomy to novel potential clinical applications.

## The Clinical Indication of CCT in the Management of Cardiomyopathies

### Coronary Anatomy Evaluation

**Figure 1:**
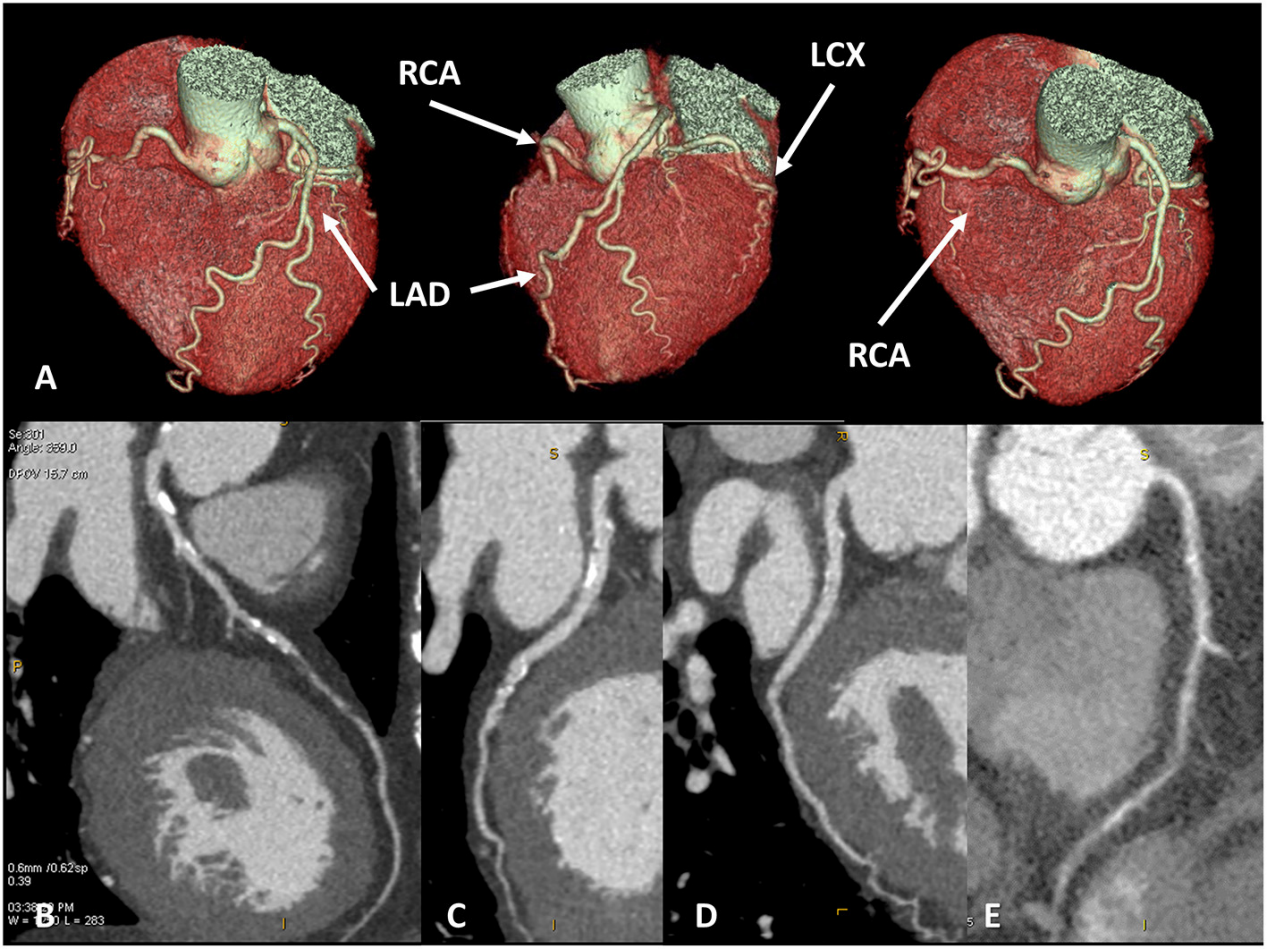
Coronary artery anatomy evaluation. A case example of 65 years old female patients with newly diagnosed severe reduction of left ventricular ejection fraction (<30%), symptomatic for dyspnea. Cardiac CT was performed with optimal image quality **(A)**. A severe multivessel coronary artery disease was found with a subocclusive lesion on mid-left anterior descendent artery **(B)**, significative lesion on left-circumflex artery **(C)**, no significative lesion on the marginal branch and subocclusive disease on right coronary artery **(D,E)**.

In the diagnostic pathway of dilated cardiomyopathy (DCM) of unknown etiology, the identification of patients with ischemic heart disease (IHD) is pivotal (9). Despite invasive coronary angiography (ICA) is still considered in patients with DCM and intermediate-to-high pretest probability of coronary artery disease (CAD) (10), CCT could be a reasonable alternative to rule out ischemic etiology when revascularization is not expected. Recently, an emerging role of coronary computed tomography angiography for preprocedural planning in those with IHD before percutaneous coronary revascularization was showed (11). Among patients with heart failure (HF) and reduced ejection fraction (HFrEF), the very high diagnostic accuracy of CCT vs. ICA for the identification of severe coronary stenosis was described, even with old generation CT (12) (Figure 1). A possible explanation of the excellent diagnostic accuracy reported is that these patients usually have an optimal heart rate as medical therapy, for HF contemplates a target heart rate <65 bpm; moreover, severe systolic dysfunction is associated with reduced cardiac and coronary motion, further improving the quality of the image. For these reasons, a consensus document from the European Society of Cardiology (ESC) published in 2019 suggested CCT as a highly valuable tool to exclude significant CAD in patients with newly diagnosed DCM (13). Similarly, ESC Guidelines on chronic coronary syndrome suggested CCT as an alternative to ICA in patients with newly diagnosed reduction of ejection fraction to establish the presence and extent of CAD and evaluate clinical indication to myocardial revascularization (13).

### Cardiac Vein Anatomy Evaluation

**Figure 2:**
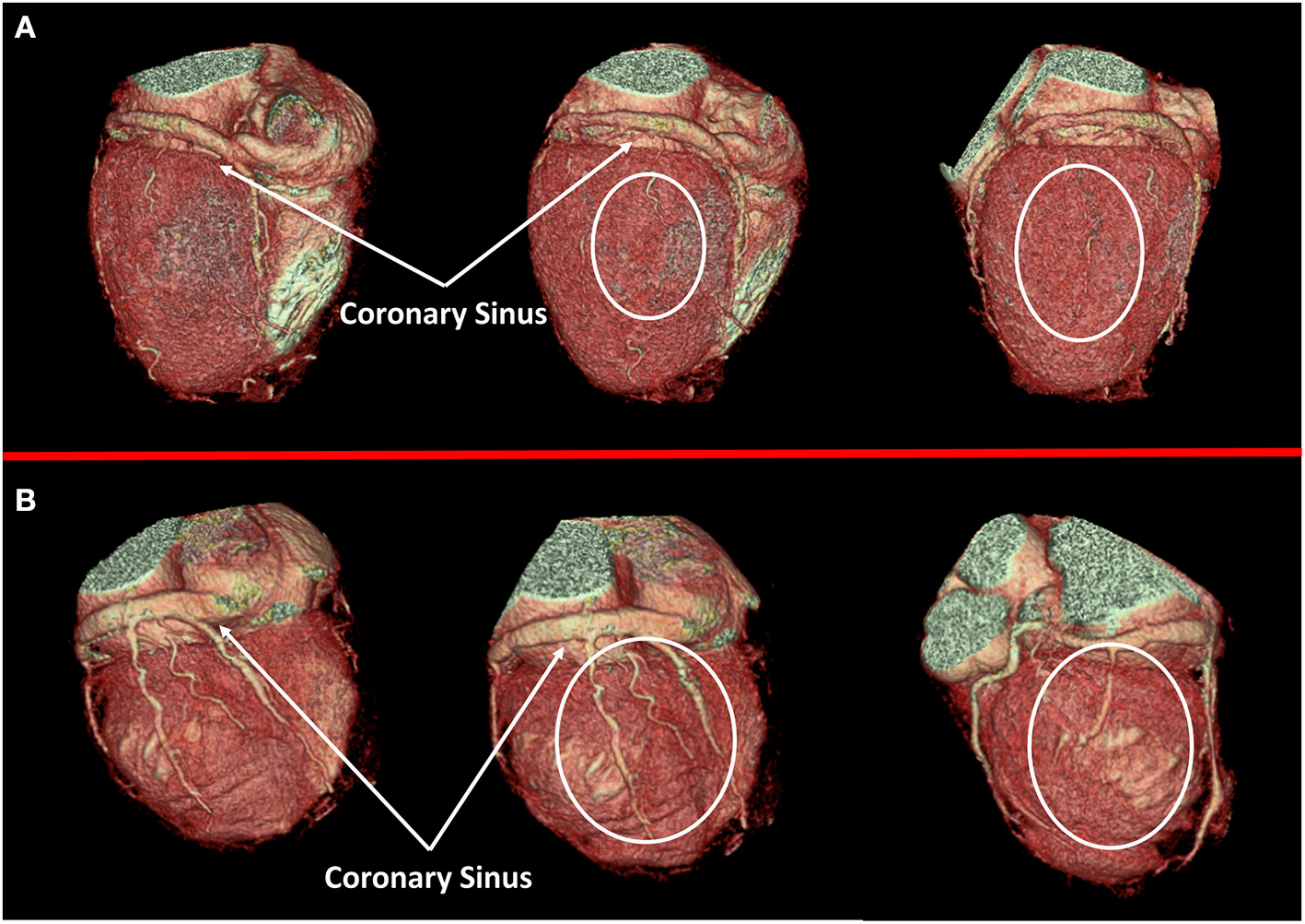
Cardiac veins anatomy evaluation. In **(A)** a case of dilated cardiomyopathy (DCM) without favorable cardiac vein anatomy for cardiac resynchronization therapy (CRT) implantation is represented; a white circle highlights the absence of adequate cardiac vein on a posterolateral wall. On the contrary, a case of optimal cardiac vein anatomy for CRT implantation is reported in **(B)**.

In 2012, Malagò et al. reported optimal imaging quality focused on cardiac vein anatomy evaluation in a consecutive cohort of patients (301 subjects) who previously underwent CCT for coronary anatomy evaluation and were retrospectively evaluated for cardiac vein mapping (14). Of interest, the authors reported an elevated variability of cardiac vein anatomy evaluated using CCT in patients with HFrEF, mostly involving the posterolateral and marginal left ventricular vein that are commonly used for cardiac resynchronization therapy (CRT). In this regard, Pontone et al. described the potential use of a dedicated acquisition protocol for cardiac vein anatomy evaluation with CT reporting an improved evaluability of a cardiac vein, especially in those with HFrEF of ischemic origin (15). In the clinical setting of patients with HFrEF, accurate characterization of cardiac vein anatomy could be of clinical interest for appropriate selection of patients with favorable anatomy before left ventricle (LV) electro-catheter implantation for CRT (16). Nowadays, after several pieces of evidence of accuracy and feasibility, non-invasive coronary vein mapping with CCT prior to placement of biventricular pacemaker is supported by clinical guidelines and consensus paper (17) (Figure 2).

*Take-home message:*

- *Cardiac computed tomography is indicated in patients with DCM of unknown etiology to rule out IHD as an alternative to ICA*
- *Cardiac computed tomography for coronary anatomy evaluation provides concomitant cardiac vein mapping in patients with HFrEF*

## An Emerging Application of CCT

### Biventricular Volumes and Systolic Function

Besides the well-validated and widely recognized role of CCT as a non-invasive tool for coronary anatomy evaluation, clinical and research interest is focusing on the potential application of CCT for a comprehensive cardiac assessment.

In 2004, Juergens et al. reported a very early experience of left ventricular volumes and systolic function evaluation with 4-slices CT compared with CMR in 30 subjects showing a good correlation between the two techniques (18). Similarly, in 2006, Sugeng et al. reported that 16-slices CCT scan provides highly reproducible measurements, especially if compared with three-dimensional echocardiography, with mild but significant overestimation of volumes and underestimation of ejection fraction when compared with CMR (19). Then, the advent in the clinical field of 64-slices CT enables concomitant right and left ventricular volumes quantification, and several reports provided evidence of the feasibility and accuracy of CT vs. CMR (20–28). A potential explanation of these discrepancies between imaging modalities could be the different respiratory phases at which images are acquired. In 2012, a meta-analysis and systematic review including 12 studies, assessing CCT-based ejection fraction obtained with 64-slices CT or dual-source CT compared with CMR or TTE (29), reported excellent concordance between CCT and MRI, especially when dual-source CCT is used; thanks to its higher temporal resolution.

**Figure 3:**
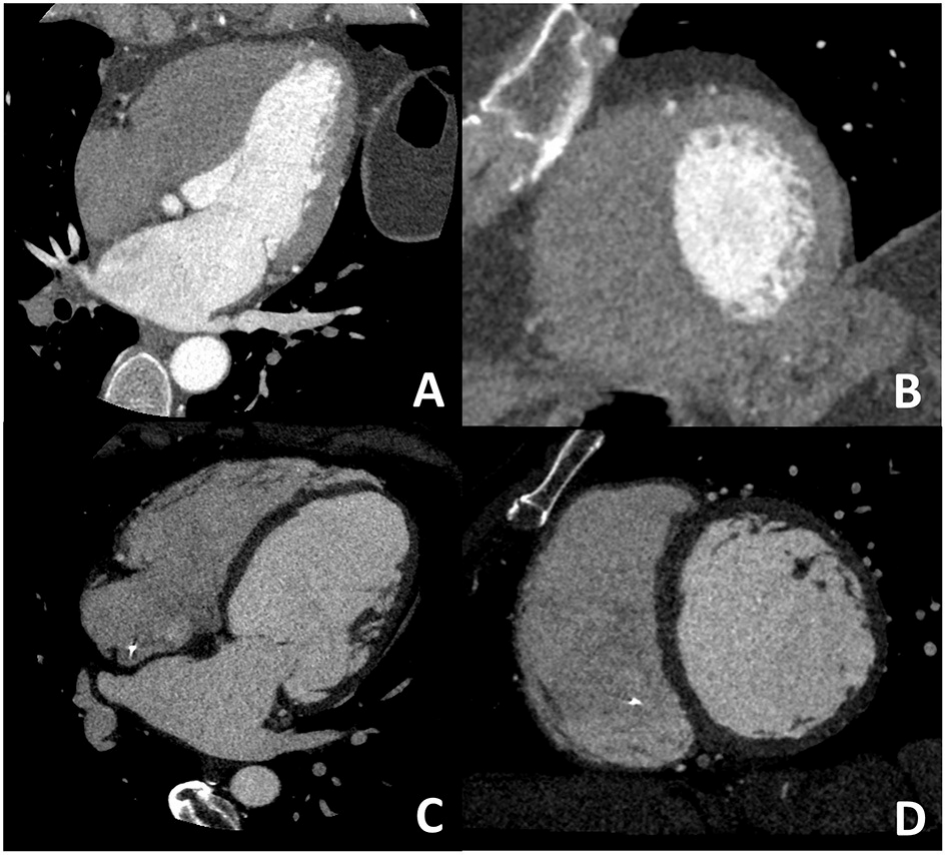
Dedicated cardiac CT (CCT) scan protocol for the comprehensive evaluation in DCM patients. In **(A,B)** a cardiac CT dedicated to coronary anatomy evaluation is shown; it is well evident how it is not possible to correctly identify the right ventricular border of the interventricular septum. In **(C,D)** a CT with dedicated acquisition protocol for biventricular volume and function in which all four chambers are opacified by contrast medium enabling correct right ventricular border identification. These examples highlight the need for a dedicated acquisition protocol for most of the non-coronary cardiac CT findings.

Despite several reports on the feasibility and accuracy of CCT for biventricular systolic function evaluation, its clinical application is limited mainly due to the elevated radiation dose up to 18–20 mSv with old-generation CT scans (30, 31). Indeed, in order to quantify ejection fraction, the entire cardiac cycle should be acquired for correct identification of end-diastolic/end-systolic phases; moreover, for the identification of the right ventricular endocardial border, a higher dose of iodinated contrast is needed to obtain a balanced contrast opacification between left and right ventricular (Figure 3).

Recently, the myocardial strain has been proposed as a promising tool for the evaluation of left and right ventricular function, especially in patients with HF and preserved ejection fraction (HFpEF). Even if echocardiography remains the most used technique for myocardial global and regional strain evaluation, both cardiac MRI (32) and, more recently, cardiac CT (33) resulted to provide accurate global strain evaluation, thanks to its volumetric data acquisition. Of interest in 2021, a study including 50 patients undergoing both CCT and CMR demonstrated very good accuracy of CCT vs. CMR in the evaluation of global myocardial strain with feature strain technique providing a true three-dimensional evaluation of all myocardial points in all the cardiac phases and in all directions (34).

In summary, despite promising results, the clinical use of CCT for biventricular volume and function is still a matter of debate especially because the need for dedicated acquisition protocol has the following three main consequences: (1) elevated radiation dose; (2) higher dose of contrast medium is usually administered for biventricular balanced contrast opacification during the entire images acquisition; (3) a *post hoc* analysis for biventricular volume and systolic function is not feasible if CT scan acquisition was focused on coronary anatomy evaluation. All these points are now limiting the clinical use of CCT for the evaluation of biventricular function. However, the promising role of CCT for the evaluation of biventricular function is confirmed by the inclusion of this technique as a potential alternative to CMR in several consensus documents oncardiomyopathies (35, 36).

**Figure 4:**
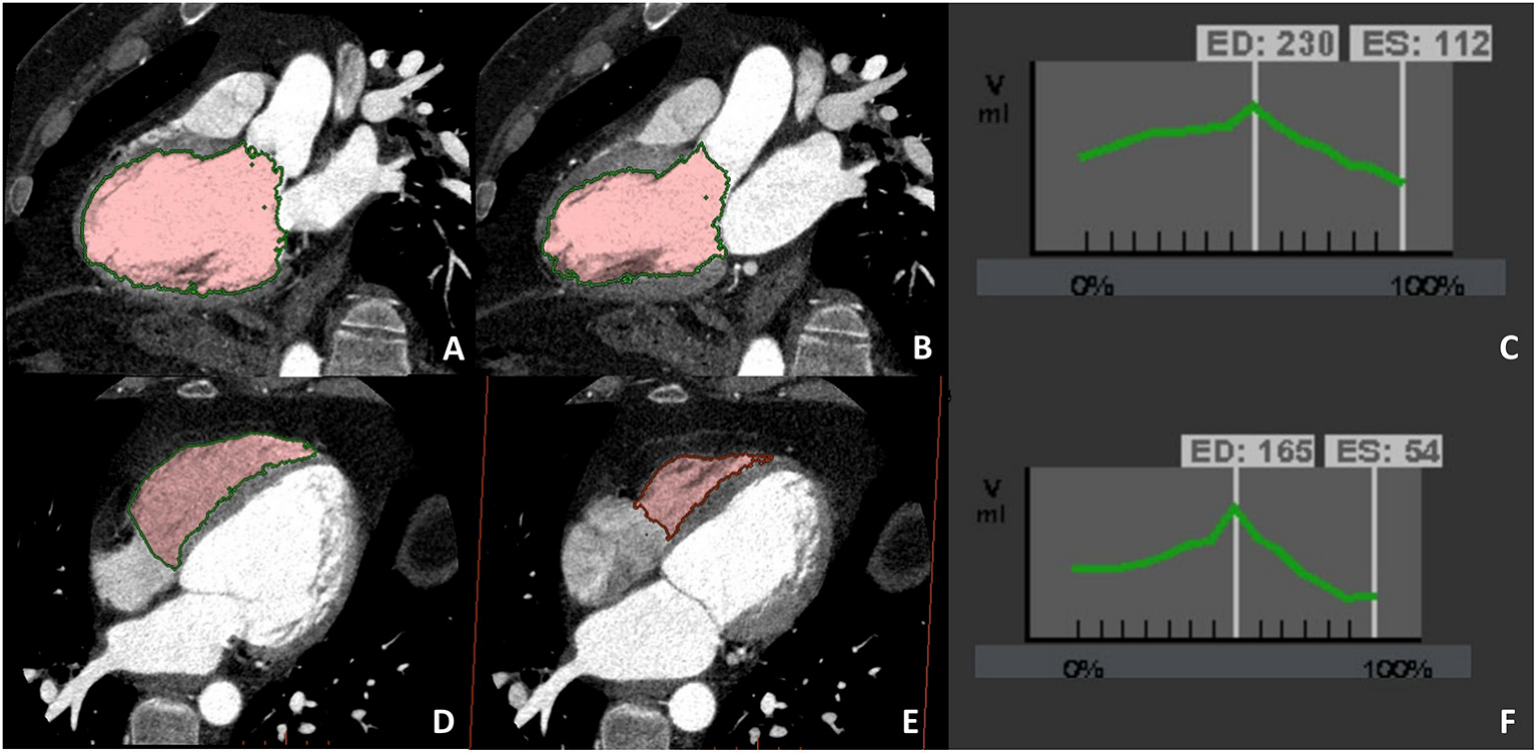
A case example of patients in which biventricular volume and function have been evaluated at CCT due to cardiac magnetic resonance (CMR) contraindication (claustrophobia). Biventricular balanced opacification enabled to correctly quantify both the left (panel **A–C**) and right (panel **D–F**) ventricular ejection fraction (LVEF 49%, RVEF 68%). Images were acquired during the entire cardiac cycle and the radiation dose needed for this evaluation was 7.5 mSv, heightening one of the main limitations to the routine clinical use of CCT for left ventricular evaluation.

Recently, the advent in the clinical field of new generation CT scans, from dual-source scanners to wide detectors enabling the entire heart volume to be covered in one beat, represents an interesting opportunity to overcome previous limitations of CCT. Moreover, new generation CT scans are characterized by a reduction in gantry rotation time that is associated with increase temporal resolution improving end-systolic/end-diastolic phase identification (Figure 4).

Results from the E PLURIBUS study (37) will soon provide important insight on this topic. The study is aiming to verify the feasibility and accuracy of single-step evaluation of biventricular volume and function, myocardial fibrosis (vs. CMR), and coronary and vein anatomy using the last generation CT scanner with a 16-cm wide detector enabling the entire cardiac volume to be acquired in a single R-R cycle; this would provide a reduction in radiation dose and contrast medium needed to obtain biventricular homogenous contrast opacification.

### Myocardial Tissue Characterization

Beyond myocardial function evaluation, myocardial fibrosis identification is widely considered to have a significant prognostic value. In 2016, a meta-analysis including 19 studies and 2,850 patients for a total of 423 arrhythmic events (38) supported the prognostic value of LGE in patients with severe reduction of ejection fraction, irrespective of the ischemic or non-ischemic nature; these data support the role of LGE for better identification of patients who may merit implantable cardioverter defibrillator (ICD) implantation. These data have been recently confirmed by the DERIVATE registry including more than 1,500 non-ischemic DCM in which a composite clinical and CMR-based risk score provides incremental prognostic value beyond the standard of care evaluation for major adverse arrhythmic cardiac events (39). Of interest, AHA/ACC 2020 guidelines on diagnosis and treatment of hypertrophic cardiomyopathy (HCM) included LGE presence and extension among parameters that should be evaluated when considering ICD implantation for primary prevention (35). In the same document, CCT is proposed as an alternative technique for an appropriate definition of LV structure including myocardial tissue characterization. These clinical recommendations are supported by some reports highlighting the capability of CCT to adequately assess myocardial structure (40). It is of the utmost importance to recognize that even CCT images acquired for coronary anatomy evaluation are suitable for myocardial thickness quantification and the identification of myocardial fat infiltration (41–43). To observe and describe these non-coronary but cardiac findings is mandatory, even if a definite diagnosis should be performed with CMR when feasible.

**Figure 5:**
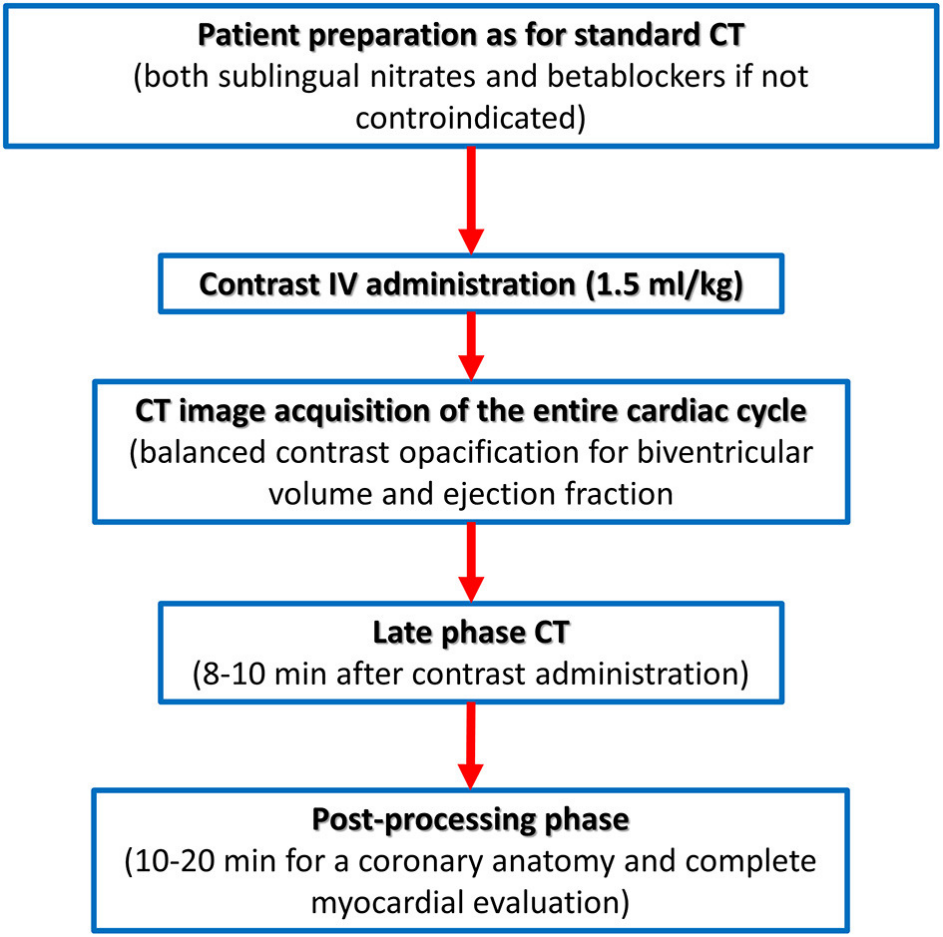
Computed tomography acquisition protocol dedicated to biventricular myocardial function and tissue characterization.

On the contrary, the recently described capability of CCT to identify the presence of myocardial fibrosis needs a dedicated acquisition protocol (Figure 5). The pathophysiological basis of myocardial fibrosis identification by contrast CCT is the same as CMR taking into consideration that pharmacokinetic properties of iodinated contrast medium are similar to gadolinium (44). The acquisition protocol for the delayed enhancement imaging in CT is nowadays based on two key rules: (1) the administration of larger amounts of iodinated contrast medium (at least 1.5 ml/kg) when compared to the dose needed for coronary anatomy evaluation and (2) the acquisition of CT images with ECG gating after 8–10 min postcontrast administration.

One of the first reports of exploring the assessment of myocardial viability was described in a small experimental study involving 17 animals (10 dogs and 7 pigs) in which an accurate identification and quantification of myocardial fibrosis by CT vs. postmortem autopsy evaluation was demonstrated (45).

In 2005, a first-in-human study enrolled 28 consecutive patients with a previous history of myocardial infarction who underwent both CMR and CCT with late CT scan for myocardial fibrosis evaluation (46); using 16-slice CT, authors reported an excellent agreement of myocardial infarction (MI) size for late-phase CCT and CMR with 415 myocardial segments (92.63%) on 448 assessed showing concordant results between the two techniques.

In 2008, le Polain de Waroux et al. enrolled 71 consecutive patients with new-onset of left ventricular dysfunction (LVD) who underwent ICA, CMR, and CCT with myocardial fibrosis evaluation (47); on a per-patient basis, CCT for both the coronary anatomy and myocardial fibrosis assessment had an excellent agreement (*k* = 0.89; *P* = 0.001) with ICA and CMR for etiological classification of LVD. For what concern myocardial fibrosis identification only two patients had false-negative results at CCT vs. CMR, possibly caused by the poor image quality and low signal-to-noise ratio. However, an overall very good accuracy (*k* = 0.88, *p* < 0.001) for the identification of fibrosis by CCT was reported.

**Figure 6:**
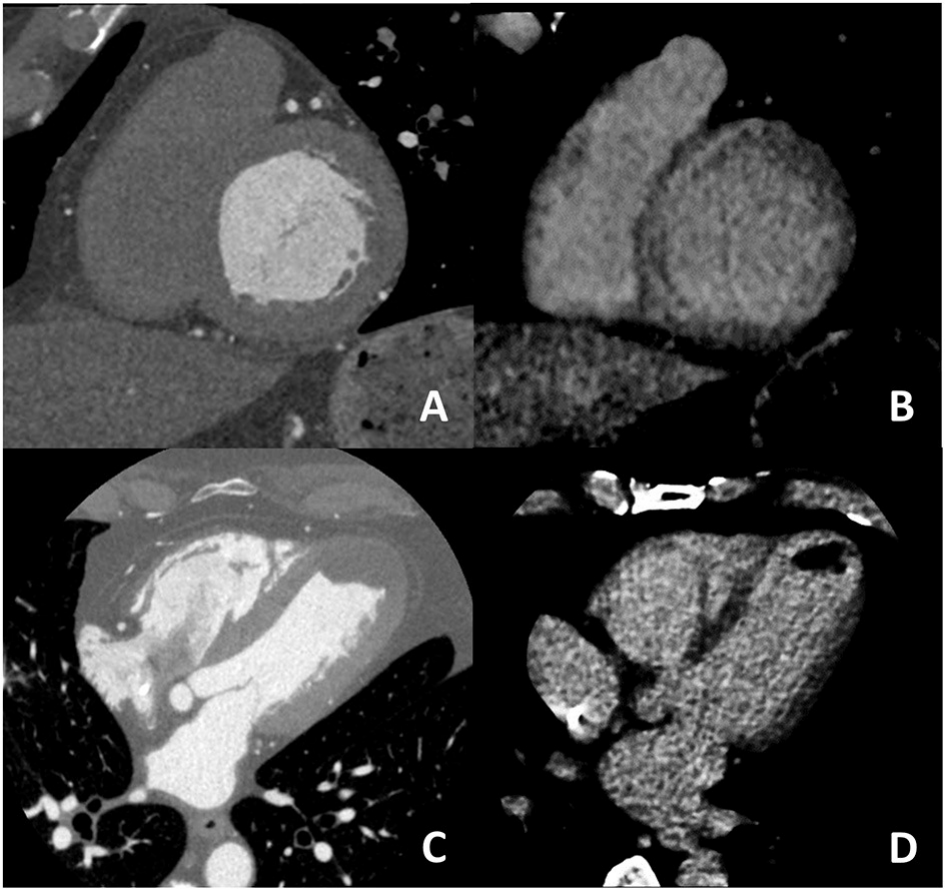
In **(A,B)** an example of non-ischemic fibrosis on the interventricular septum is shown; more specifically, in **(A)** angiographic phase CT is presented with corresponding late CT acquisition well demonstrating non-ischemic mid-wall myocardial fibrosis in **(B)**. In **(C,D)** a case of ischemic cardiomyopathy is presented; an apical left ventricular thrombus is evident both at first pass CT **(C)** and at late phase CT **(D)** from which the ischemic subendocardial fibrosis could be evidenced.

For what concern non-ischemic fibrosis, a validation study was performed in 2014 including 24 patients with hypertrophic phenotype who underwent both CCT and CMR for myocardial fibrosis evaluation (Figure 6). On a per-patient basis, CCT had a 100% sensitivity for myocardial fibrosis vs. CMR and mean myocardial scar area resulted of 2.2 ± 1.4 cm^2^ in CCT vs. 2.9 ± 2.4 cm^2^ in CMR; of interest, authors reported that the relative intensity ratio between normal remote myocardium and area of myocardial fibrosis in CT was 1.8 ± 0.3 (48). Similar promising results have been recently reported in a consecutive cohort of patients with sarcoidosis evaluated with both CCT and CMR (49). For optimal evaluation of myocardial fibrosis at late CT scan, dedicated post-processing analysis is needed, as previously described (50). More precisely, an 8–10 mm thickness in short-axis LV view should be obtained and narrow window and level settings (W300, L150) are suggested (Figure 5).

Despite promising results, myocardial fibrosis evaluation by CCT remains limited by low signal-to-noise ratio and by the need for a high dose of contrast medium. Technological advances are needed to overcome these limitations. One of the most promising novelties in this field is the use of dual-energy CT that appeared to enable myocardial fibrosis identification with reduced contrast amount. Dual-energy CT (both with dual-source CT or with single-source CT and rapid kVp switching) technology permits contemporary use of different tube potential enabling tissue characterization with extracellular volume (ECV) estimation that is considered a myocardial fibrosis equivalent when evaluation with CMR. More precisely, in 2020, Ohta et al. reported that strong correlations were seen between CT-ECV and MR-ECV at postcontrast CT images in 23 patients (51). Similarly, in 2019 Kumar et al. (52) reported that multi-energy CT in 21 subjects, when compared with a single-energy approach, better discriminate the presence or absence of myocardial fibrosis severity when compared with CMR, with correct classification rates of 89 and 71%, respectively; similarly, the multi energy CT better discriminates normal from elevated ECV, with a correct classification in 89% of patients vs. correct distinction of normal vs. elevated ECV in only 70% using single energy CT. Recently, radiomics models with an artificial intelligence approach achieved a good diagnostic accuracy (AUC: 0.78, 95% CI: 0.75–0.81) on a per-segment basis for the identification of myocardial fibrosis with CCR vs. MRI (53). Even if promising, these novel approaches need further studies before being proposed for clinical use.

*Take-home messages:*

- *Biventricular volume and systolic function analysis are feasible and accurate with CCT*.
- *Myocardial fibrosis assessment is feasible with CT, even if a low contrast-to-noise ratio limits diagnostic accuracy vs. CMR*.
- *For both the myocardial fibrosis and biventricular function assessment with CT, dedicate acquisition protocol is needed with an increase in radiation dose and iodinated contrast amount administration, limiting their clinical use to those patients with CMR contraindication*.
- *In the next future, technological advances may further expand the clinical application of non-coronary CCT evaluation*.

## Technical Consideration for a Comprehensive Approach

As previously outlined, a dedicated acquisition protocol for comprehensive evaluation of myocardial fibrosis and biventricular volumes and function is needed; it is important to underline that coronary anatomy analysis is feasible using the same imaging dataset. More precisely, biventricular volumes and function are evaluated using imaging acquired at the first pass contrast angiographic phase, the same used for coronary evaluation. What differs from traditional CCTA is the total volume of contrast needed (at least 1.5 ml/kg for function and fibrosis vs. <1 ml/kg for coronary anatomy) and the need for biventricular opacification, that may cause, in rare cases, artifacts on the mid-portion of the right coronary artery. On the other side, the need for contrast medium bolus for optimal angiographic evaluation of coronary vessels has been indicated as a potential cause to mild, but significative, overestimation of right ventricular end-diastolic volume (RVEDV); to reduce discrepancies vs. CMR in RVEDV quantification, contrast injection at lower infusion rate has been proposed, but this approach would make coronary stenosis not evaluable. Overall, high priority should be given to coronary anatomy evaluation as this is the most important and validated CCTapplication.

For what concerns myocardial fibrosis evaluation, the late post-contrast acquisition is needed without a significative increase in radiation dose. It should be underlined that, especially in patients with DCM, where a higher dose of contrast medium is needed to evaluate coronary anatomy, adding a late post-contrast scan could be proposed on a regular basis; on the contrary, this is not true for biventricular volumes and function evaluation for which the need of entire cardiac cycle acquisition is associated with a significative increase in radiation dose.

Finally, it should be underlined that all these advanced uses of cardiac CT are strictly dependent on the availability of the last generation CT; indeed, 64-slice CT technology is suboptimal especially for biventricular volumes and function analysis and is associated with very high radiation dose (up to 1,820 mSv), prohibitive especially when serial evaluation at follow-up is needed.

## Emerging Clinical Application in Specific Settings

### Hypertrophic Cardiomyopathy

Several clinical fields may benefit from a wider application of CCT beyond coronary anatomy evaluation, even if extensive supporting data are still to come. The unique capability of CCT to evaluate both coronary and myocardial anatomy may support its use in the evaluation of patients with HCM. In this setting, beyond the analysis of myocardial thickness and fibrosis, CCT may provide an accurate evaluation of the concomitant presence of coronary artery disease; more precisely, in a previous study including 60 patients with HCM (54), CCT provided a 100% sensitivity, a 94.4% specificity, a 92.3% positive predictive value, and a 100% negative predictive value for the identification of significative coronary stenosis when compared with ICA. Moreover, CCT may enable accurate evaluation of myocardial bridges, whose prevalence among patients with HCM is not negligible, and up to 40% according to previous reports (54). Previous studies suggested that myocardial bridges among patients with HCM are longer and deeper when compared to a control group of patients (55); thus, accurate evaluation of coronary anatomy is of utmost importance in these patients. Moreover, in those with clinical indications to myomectomy preprocedural planning with CCT may provide important insight on septum anatomy for a safer and more effective procedure, as previously suggested (56). Moreover, CCT may provide an accurate evaluation of right ventricular wall thickness with identification of wall hypertrophy that could be missed at TEE.

### Arrhythmogenic Cardiomyopathy

Accurate analysis of the right ventricle is of the utmost importance in patients with suspected arrhythmogenic cardiomyopathy (AC); even if validated cut-offs for AC diagnosis are still missing, the presence of right ventricular bulging and/or right ventricular dilation or systolic dysfunction could be identified with CCT. However, it should be underlined that previous reports suggested a mild, but significative, overestimation of right ventricular diastolic volume with CT vs. CMR (31), possibly because of the different phases of the respiratory cycle at which images are obtained in the two techniques and/or to the contrast medium bolus that is administered during CT images acquisition. Of interest, the recent data correlated right ventricle wall tissues heterogeneity identified at CT with abnormal findings at invasive electroanatomical mapping (EAM), proposing CT as a potential tool for accurate identification of patients with AC vs. those with right ventricular dilation due to adaptive remodeling (i.e., athletes) (57).

### Preprocedural Planning of Ventricular Arrhythmias Ablation

For what concerns myocardial tissue analysis, one of the most promising clinical applications of CCT is the evaluation of anatomical substrate in patients with unstable ventricular arrhythmias and possible indications to transcatheter ablation. In this setting, identification of myocardial arrhythmic substrate by CMR has been demonstrated to improve procedural outcome (58); unfortunately, CMR could sometimes be of difficult feasibility due to both the presence of unstable heart rhythm (with safety issues of potential patients in an MRI environment) and previous implanted electronic device (i.e., intern defibrillator). A recently published state-of-the-art paper well-summarized clinical indication and acquisition protocol for CCT in this specific clinical setting (59); the most interesting feature is the possibility to import CCT data during invasive electrophysiological mapping providing live guidance based on anatomical substrate and possibly avoid ICA. More precisely, CCT enables concomitant myocardial tissue characterization (myocardial fibrosis) and analysis of coronary anatomy, excluding severe coronary stenosis as the cause of arrhythmic storm; both these information can be imported during the invasive electrophysiological procedure for live visualization and guidance. Moreover, a detailed analysis of the anatomy at the access site for the epicardial approach could be performed for optimal preprocedural planning. Esposito et al. reported that CCT with late scan acquisition was able to detect myocardial scars responsible for pathological low-amplitude voltages at invasive electroanatomic mapping with high sensitivity and very high negative predictive values (76 and 95%, respectively), regardless of substrate etiology and ICD presence (60).

Apart from this advanced application, CCT should be considered in clinical practice for biventricular volume and function analysis and myocardial fibrosis identification when CMR is contraindicated and TTE is inconclusive. This approach is supported by guidelines and consensus documents previously published (9, 17, 35, 36, 61).

## Limitation of Novel Clinical Application of CCT

Despite promising results from CCT, nowadays, CMR remains the gold standard for biventricular volume and function quantification and for myocardial tissue characterization, as several technical reasons limit CCT clinical application for non-coronary evaluation. First, the temporal resolution of CCT is lower when compared with CMR. Second, CCT analysis is associated with non-negligible radiation dose (at least 5–6 mVs for a complete examination including coronary anatomy, biventricular volumes/function, and myocardial fibrosis); this is of particular concern in young patients who may undergo serial CCT during follow-up and for which cumulative radiation dose would result to be prohibitive. Moreover, the diagnostic accuracy of CCT for myocardial fibrosis evaluation is lower when compared to CMR due to a lower signal-to-noise ratio.

These limitations could be overcome in the future if further technological advances, especially in the field of artificial intelligence, should be applied in clinical practice (62).

## Conclusion

When CMR is contraindicated and TTE results to be inconclusive, CCT may be considered in clinical practice as an alternative to CMR during diagnostic evaluation of patients with cardiomyopathies (Table 1). Adequate CT technology (preferable more than a 64-slice scanner) and a dedicated acquisition protocol are needed for biventricular function assessment and myocardial fibrosis evaluation. Of note, non-coronary but cardiac findings should be always carefully assessed, as myocardial hypertrophy and fat infiltration are often well evident even if CCT is focused on coronary anatomy.

**Table 1:** Main pro/cons features of CCT compared with other imaging modalities according to different clinical settings.

	**Transthoracic echocardiography**		**Cardiac MRI**		**Cardiac CT**	
	**Pro**	**Cons**	**Pro**	**Cons**	**Pro**	**Cons**
All settings	Wide availability	Tissue characterization is not feasible	Well-validated tissue characterization	Dedicated environment may represent contraindication	Non-invasive evaluation of coronary anatomy	Radiation dose, especially if serial evaluation needed at follow-up
DCM	Well-validated prognostic role of ejection fraction in this setting	Apical thrombus could be missed	Enable differential diagnosis among non-ischemic etiologies (i.e., myocarditis)	Diastolic function evaluation is not feasible	Biventricular function analysis and coronary anatomy evaluation is feasible at the same time	Higher dose of contrast medium is needed due to chamber dilation
HCM	Enable dynamic evaluation of hemodynamic features (LVOT obstruction)	Apical hypertrophy could be missed	Myocardial fibrosis identification has well-validated prognostic role	Hemodynamic evaluation is not feasible	Identification of myocardial bridging and coronary artery disease	Hemodynamic evaluation is not feasible
ARVC	Comprehensive evaluation of right ventricular hemodynamic pattern	Right ventricular evaluation could be suboptimal in some cases	Accurate analysis of right ventricular kinesis, volumes and function	Accurate evaluation of right ventricular could be difficult due to frequent ventricular arrhythmias	Enable exclusion of other causes of right ventricular dilation	Possible over-estimation of right ventricular end-diastolic volume

## Author Contributions

EC and SM wrote the first draft of the manuscript. GM, AF, AA, EMe, and FR performed literature research and data evaluation. EMa, CG, ZL, MG, AB, RM, and CMG performed manuscript and figure editing and revision. CC, VC, MP, DA, and GP provided expert revision and supervision. All authors contributed to the article and approved the submitted version.

## Funding

Centro Cardiologico Monzino will cover publication fees.

## Conflict of Interest

The authors declare that the research was conducted in the absence of any commercial or financial relationships that could be construed as a potential conflict of interest.

## Publisher's Note

All claims expressed in this article are solely those of the authors and do not necessarily represent those of their affiliated organizations, or those of the publisher, the editors and the reviewers. Any product that may be evaluated in this article, or claim that may be made by its manufacturer, is not guaranteed or endorsed by the publisher.
